# A Case of Long-undiagnosed Classic Kaposi’s Sarcoma with Extensive Skin Eruptions

**DOI:** 10.31662/jmaj.2024-0161

**Published:** 2025-01-31

**Authors:** Kouhei Takehara, Yasuhito Hamaguchi, Kou Fujii, Motoki Horii, Natsumi Fushida, Tasuku Kitano, Shintaro Maeda, Kyosuke Oishi, Katsushige Taniuchi, Takashi Matsushita

**Affiliations:** 1 Department of Dermatology, Faculty of Medicine, Institute of Medical, Pharmaceutical and Health Sciences, Kanazawa University, Kanazawa, Japan; 2 Department of Dermatology, Noto General Hospital, Nanao, Japan

**Keywords:** Kaposi’s sarcoma, HHV-8, Skin eruption, Immunohistochemical staining

## Abstract

Kaposi’s sarcoma (KS) is a chronic, multifocal lymphoangioproliferative tumor that occurs mainly in older individuals in the Mediterranean region. KS is often observed as a malignant tumor in patients with acquired immunodeficiency syndrome, and classic KS is rare. An 82-year-old man was referred to our department with bilateral lower-leg edema with purpura and small nodules. When the patient was in his mid-50s, edema with purpura appeared in his lower extremities without any specific trigger. Eight years earlier (in his mid-70s), the purpura lesions gradually turned nodular. A similar skin rash appeared on the forearms and back of the hands two years earlier. Physical examination revealed diffuse brown pitting edema over the entirety of the lower extremities and extending from the dorsum of the bilateral hands to the elbows. Multiple purple-red, elastic, hard nodules ranging from a few millimeters to about 1 cm were localized and fused over the edema. Histological examination of the forearm nodule revealed proliferation of collagen fibers and cellular infiltration in the dermis. The intricately proliferating cells in the dermis were spindle-shaped with round nuclei. Immunohistochemical staining revealed spindle-shaped cells positive for CD31, CD34, and D2-40. These cells were positive for human herpesvirus (HHV)-8 and negative for human immunodeficiency virus (HIV) antigens and antibodies. He was diagnosed with classic KS. We proposed chemotherapy, but he refused to receive any treatment. Our patient did not receive any immunosuppressive therapy, and the HIV test result was negative. Therefore, immunosuppressive status may not be involved in the development of KS in our patient. He had opportunities to consult dermatologists but was never diagnosed with KS. A skin biopsy helps diagnose KS; thus it should be considered when a patient experiences long-standing, slowly progressive, unexplained leg edema accompanied by a skin rash.

## Introduction

Kaposi’s sarcoma (KS) was first described by Kaposi in 1872 as a chronic, multifocal lymphoangioproliferative tumor that occurs mainly in the elderly population in the Mediterranean region ^(1)^. With the increase in the number of acquired immunodeficiency syndrome cases since the 1980s, KS is often observed as a malignant tumor in patients with acquired immunodeficiency syndrome, and classic KS is rare. Here, we experienced a classic type of KS with extensive skin eruptions, which had been undiagnosed for approximately 30 years.

## Case Report

**Figure 1.**
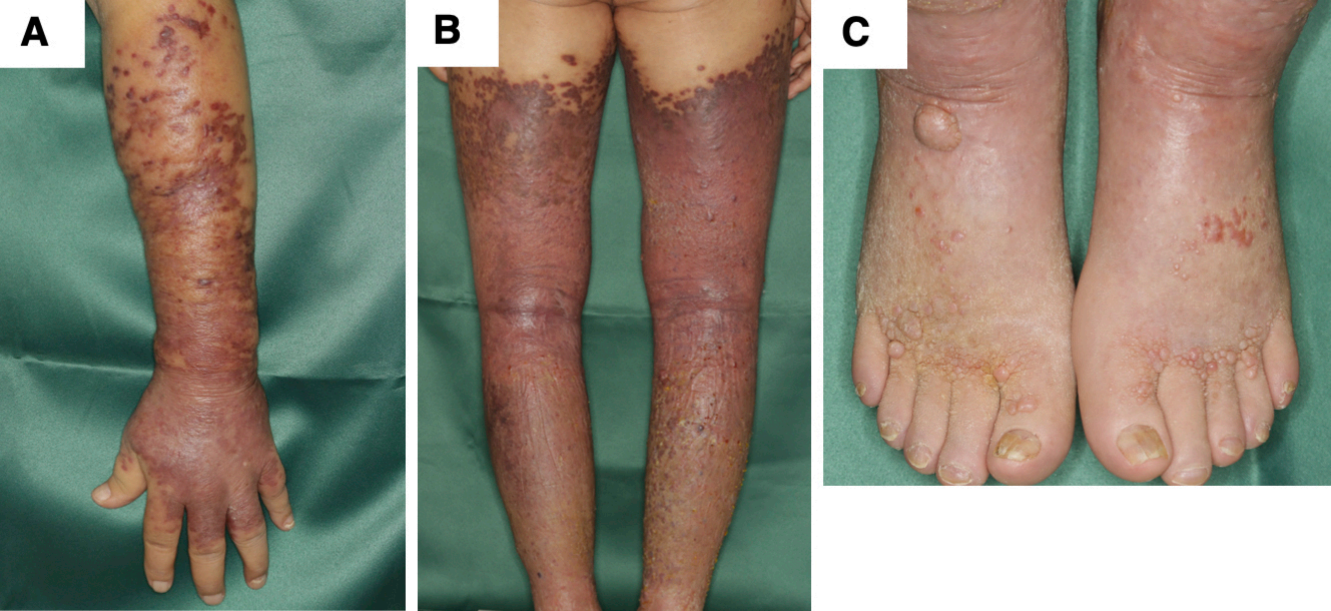
A-B. Diffuse pitting edema with multiple purple-red-brown nodules and brown plaques on the lower extremities and from the dorsum of the hand to the elbow. C. A dome-shaped nodule with a small crust at the dorsum of the foot.

**Figure 2.**
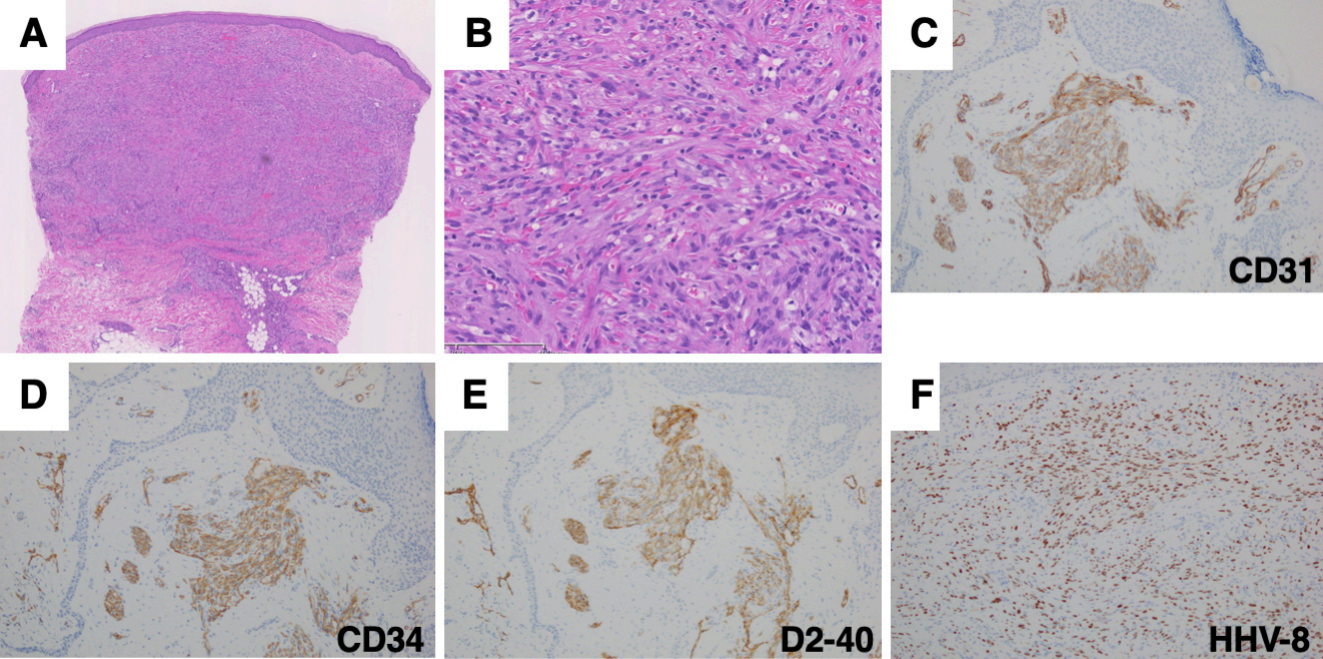
A-B. Histological findings of the nodule on the forearm. C-F. Immunohistochemical staining of CD31 (C), CD34 (D), D2-40 (E), and HHV-8 (F). Original magnification ×12.5 (C), ×200 (D), and ×100 (E-F).

An 82-year-old man was referred to our department with bilateral lower-leg edema with purpura and small nodules. He was born in China and immigrated to Japan in his mid-40s. He had been taking antihypertensive medication since his 50s. He was also diagnosed with macrocytic anemia and was taking folic acid and ferrous citrate for 2 years. When the patient was in his mid-50s, edema with purpura appeared in his lower extremities without any specific trigger. He was advised to wear elastic stockings for edema by the dermatologists several times. Eight years earlier (in his mid-70s), the purpura lesions gradually turned nodular. A similar skin rash appeared on the forearms and back of the hands 2 years earlier. He had no pain, itching, or other subjective symptoms. Physical examination revealed diffuse brown pitting edema over the entire lower extremities, extending from the dorsum of the bilateral hands to the elbows, with multiple purple-red, elastic hard nodules ranging from a few millimeters to approximately 1 cm localized and fused over the edema (Figure 1A, 1B and 1C). Purple-to-brown, slightly raised rashes were observed near the border of the large plaques. Multiple small ulcers were macerated and accompanied by yellowish-clear fluid, indicating lymph leakage. Histological examination of the nodule on the left forearm revealed proliferation of collagen fibers and cellular infiltration in the dermis (Figure 2A). The intricately proliferating cells in the dermis were spindle-shaped cells with round nuclei (Figure 2B). They contained erythrocytes with minute clefts. Spindle-shaped cells had increased amounts of nuclear chromatin, small nucleoli, and abnormal mitosis. Immunohistochemical staining revealed spindle-shaped cells positive for CD31, CD34, and D2-40 (Figure 2C, 2D and 2E). These cells were human herpesvirus (HHV)-8 positive (Figure 2F). Blood tests showed mild renal dysfunction (creatinine; 1.20 mg/dL), hypoproteinemia (total protein; 6.0 g/dL), hypoalbuminemia (albumin; 3.3 g/dL), and mild hypothyroidism. The platelet count was 21.4 × 10^4^/μL. The coagulation test results were normal. Testing for lupus anticoagulant, anticardiolipin antibodies, and cryoglobulins was negative. The serum IgG level was within normal range (1177 ml/dL). Human immunodeficiency virus (HIV) antigens and antibodies were negative. His cardiac function was normal. Internal organ involvement was not detected. He was diagnosed with classic KS. We proposed chemotherapy, but he refused to receive any treatment.

## Discussion

KS is associated with opportunistic tumors in patients undergoing immunosuppressive therapy. Our patient did not receive any immunosuppressive therapy, and the HIV test result was negative. Therefore, immunosuppressive status may not be involved in the development of KS in our patient.

KS is an endemic disease because of regional differences in HHV-8 infection rates: 50% in Africa, 15%-20% in the Mediterranean region, and 10% or lower in the Americas, northern Europe, and Asia ^(2)^. The seroprevalence of HHV-8 throughout Japan is 1.4% ^(3)^. However, even in Japan, there are significant regional differences in the incidence of KS. The age-adjusted incidence rate was 0.87/10^5^ per year for the Miyako Islands and 0.056/10^5^ per year for the rest of Okinawa ^(2)^. Similar regional differences were observed in China. The Uygur and Han populations in the Xinjiang-Uygur Autonomous Region have a similar HHV-8 seroprevalence of approximately 20%; however, KS is observed only in Uygur individuals ^(4)^. These findings indicate that genetic factors may contribute to the development of KS.

There are two treatment strategies for KS, local and systemic therapies. Chemotherapy is recommended for visceral involvement or the rapid progression of lymphedema. In addition, compression stockings may help control KS-related edema as therapeutic intervention.

Our patient had opportunities to consult dermatologists but was never diagnosed with KS. KS can be challenging to diagnose, particularly when the clinical presentation is atypical. One finding that may suggest atypical KS is the distribution of the lesions. KS lesions are generally restricted to the extremities. Therefore, KS should be considered in the differential diagnosis when a skin rash with multiple nodules is restricted to the extremities. A skin biopsy helps diagnose KS; thus, it should be performed when a patient experiences long-standing, slowly progressive, unexplained leg edema accompanied by a skin rash.

## Article Information

### Conflicts of Interest

None

### Author Contributions

Drs. Matsushita and Hamaguchi had full access to all of the data in this report and take responsibility for the integrity of the data and the accuracy of the data analysis.

Acquisition of data: Drs. Takehara, Hamaguchi, Fujii, Horii, Fushida, Kitano, Maeda, Oishi, Taniuchi, and Matsuhita.

Drafting of the manuscript: Drs. Takehara, Hamaguchi, and Matsushita.

All authors have read and approved the final manuscript.

### Informed Consent

Written informed consent was obtained from the patient prior to the publication of this case report.
